# Supplementary material for: An epigenetic switch regulates the ontogeny of AXL-positive/EGFR-TKi-resistant cells by modulating miR-335 expression
Source: eLife. 2021 Jul 13;10:e66109. doi: 10.7554/eLife.66109 (PMC8285107; doi:10.7554/eLife.66109)
Supplement: Supplementary file 1. [file elife-66109-supp1.docx]

| **Primers for quantitative real-time PCR analysis of miRNAs** | | |
| --- | --- | --- |
| **MicroRNA PCR primer set** | **Target sequence** | **EXIQON Product No.:** |
| Downregulated microRNA’s | | |
| hsa-let-7c | UGAGGUAGUAGGUGGUAUGGUU | 204767 |
| hsa-miR-17 | CAAAGUGCUUACAGUGCAGGUAG | 204771 |
| hsa-miR-18a | UAAGGUGCAUCUAGUGCAGAUAG | 204207 |
| hsa-miR-20a | UAAAGUGCUUAUAGUGCAGGUAG | 204292 |
| hsa-miR-29c | UAGCACCAUUUGAAAUCGGUUA | 204729 |
| hsa-miR-31 | AGGCAAGAUGCUGGCAUAGCU | 204236 |
| hsa-miR-34a | UGGCAGUGUCUUAGCUGGUUGU | 204486 |
| hsa-miR-92a | UAUUGCACUUGUCCCGGCCUGU | 204258 |
| hsa-miR-95 | UUCAACGGGUAUUUAUUGAGCA | 204288 |
| hsa-miR-99a | AACCCGUAGAUCCGAUCUUGUG | 204521 |
| hsa-miR-141 | UAACACUGUCUGGUAAAGAUGG | 204504 |
| hsa-miR-182 | UUUGGCAAUGGUAGAACUCACACU | 204264 |
| hsa-miR-183 | UAUGGCACUGGUAGAAUUCACU | 204652 |
| hsa-miR-192 | CUGACCUAUGAAUUGACAGCC | 204099 |
| hsa-miR-194 | UGUAACAGCAACUCCAUGUGGA | 204080 |
| hsa-miR-200c | UAAUACUGCCGGGUAAUGAUGG | 205112 |
| hsa-miR-205 | UCCUUCAUUCCACCGGAGUCUG | 204487 |
| hsa-miR-224 | CAAGUCACUAGUGGUUCCGUUUA | 205121 |
| hsa-miR-335 | UCAAGAGCAAUAACGAAAAAUGU | 204151 |
| hsa-miR-452 | UGUUUGCAGAGGAAACUGAGAC | 205074 |
| **Upregulated microRNA’s** | | |
| hsa-let-130b | CAGUGCAAUGAUGAAAGGGCAU | 204317 |
| hsa-miR-137 | UUAUUGCUUAAGAAUACGCGUAG | 204655 |
| hsa-miR-143 | UGAGAUGAAGCACUGUAGCUC | 204190 |
| hsa-miR-152 | UCAGUGCAUGACAGAACUUGG | 204294 |
| hsa-miR-153 | UUGCAUAGUCACAAAAGUGAUC | 204338 |
| hsa-miR-181c | AACAUUCAACCUGUCGGUGAGU | 204583 |
| hsa-miR-193a | UGGGUCUUUGCGGGCGAGAUGA | 204665 |
| hsa-miR-195 | UAGCAGCACAGAAAUAUUGGC | 204186 |
| hsa-miR-218 | UUGUGCUUGAUCUAACCAUGU | 204484 |
| hsa-miR-221 | AGCUACAUUGUCUGCUGGGUUUC | 204532 |
| hsa-miR-328 | CUGGCCCUCUCUGCCCUUCCGU | 204364 |
| hsa-miR-486-5p | UCCUGUACUGAGCUGCCCCGAG | 204001 |
| hsa-miR-497 | CAGCAGCACACUGUGGUUUGU | 204354 |
| hsa-miR-532-5p | CAUGCCUUGAGUGUAGGACCGU | 204221 |
| hsa-miR-660 | UACCCAUUGCAUAUCGGAGUUG | 204727 |
| U6 snRNA |  | 203907 |

| **Primers for quantitative real-time PCR** | | |
| --- | --- | --- |
| **Target** | **Forward (5’-3’)** | **Reverse (5’-3’)** |
| ACTIN | CATGTACGTTGCTATCCAGGC | CTCCTTAATGTCACGCACGAT |
| ARPC2 | CTGGAGGTGAACAACCGCAT | GACCCCATCGAAATCTGCAAA |
| AXL | GACCGGCCAAGTTTTACAGA | ATAACCTCCACCCTCATCCA |
| CAV1 | AATACTGGTTTTACCGCTTGCT | CATGGTACAACTGCCCAGATG |
| CAV2 | AAGAACTGCCTAATGGTTCTGC | CTCGTACACAATGGAGCAATGAT |
| CD109 | CTGATGGCAACCAACTGACTC | TTCCACTGTTAGATCCGCTCC |
| CD24 | CTCCTACCCACGCAGATTTATTC | AGAGTGAGACCACGAAGAGAC |
| CD44 | TTGCAGTCAACAGTCGAAGAAG | CCTTGTTCACCAAATGCACCA |
| ECAD | CGAGAGCTACACGTTCACGG | GGCCTTTTGACTGTAATCACACC |
| EGFR | AGGCACGAGTAACAAGCTCAC | ATGAGGACATAACCAGCCACC |
| GAPDH | GGCTGAGAACGGGAAGCTTGTCA | CAGCCTTCTCCATGGTGGTGAAGA |
| GAS6 | GTTCTCCTGGCTGCATTCGTTGA | CATCAACAAGTATGGGTCTCCGT |
| MMP14 | GGCTACAGCAATATGGCTACC | GATGGCCGCTGAGAGTGAC |
| MSN | GAGGATGTGTCCGAGGAATTG | GTCTCAGGCGGGCAGTAAA |
| PRNP | AGTCAGTGGAACAAGCCGAG | ACCAGCCATGTGCTTCATGT |
| PTRF | GGGCCGTAGACCAGATCCA | CTTGCTCACCGTATTGCTCGT |
| RRAS2 | TAAAGGATCGTGATGAGTTCCCA | AATTTCCTGATAACCCGGACAAG |
| SNAIL | TCGGAAGCCTAACTACAGCGA | AGATGAGCATTGGCAGCGAG |
| SLUG | AAGCATTTCAACGCCTCCAAA | GGATCTCTGGTTGTGGTATGACA |
| THBS1 | AGACTCCGCATCGCAAAGG | TCACCACGTTGTTGTCAAGGG |
| SOX9 | AGCGAACGCACATCAAGAC | CTGTAGGCGATCTGTTGGGG |
| TGFβ1 | GGCCAGATCCTGTCCAAGC | GTGGGTTTCCACCATTAGCAC |
| TGFβ2 | TTTGGTGAAAGCAGAGTTCAGAGTC | ATGTAGCGCTGGGTTGGAGA |
| TTF1 | GACCTCAGAAACATTCCCACG | AAGTGGCATTTGCAGTCTAT |
| VIM | AGAACTTTGCCGTTGAAGCTG | CCAGAGGGAGTGAATCCAGATTA |
| WNT5a | ATTCTTGGTGGTCGCTAGGTA | CGCCTTCTCCGATGTACTGC |
| XBP1 | AGTAGCAGCTCAGACTGCCA | CTCACTCATTCGAGCCTTCTTT |
| ZEB1 | GCACCTGAAGAGGACCAGAG | TGCATCTGGTGTTCCATTTT |

| **Primers for methylation specific PCR** | | | |
| --- | --- | --- | --- |
| **Target** | | **Forward (5’-3’)** | **Reverse (5’-3’)** |
| MEST  CpG island 1 | Methylated | GGTTGAAATACGGAAGGTTC | AACAAACCGTAACTTCGACA |
|  | Unmethylated | AAGGGTTGAAATATGGAAGGTTT | AACAAACCATAACTTCAACAATA |
| MEST  CpG island 2 | Methylated | GATTTAGGTATTACGGTGGC | TCACTCGCGCTTATTACTAA |
|  | Unmethylated | TAAGATTTAGGTATTATGGTGGT | CTCACTCACACTTATTACTAAAA |
| **Cloning primers for bisulfide sequencing** | | | |
| **Target** | | **Forward (5’-3’)** | **Reverse (5’-3’)** |
| MEST CpG island 1 | | ATGGATATTATTTTTAGGGTAATTG | CCTTACCTACAAAACTCCATATT |
| MEST CpG island 2 | | TGTAGTTTAGGATTTTAAGATTTAGGT | TTCCAAAAATTTCTAAACCTTAA |
